# Recovery and Regeneration of Spent Lithium-Ion Batteries From New Energy Vehicles

**DOI:** 10.3389/fchem.2020.00807

**Published:** 2020-10-29

**Authors:** Qing Zhao, Lv Hu, Wenjie Li, Chengjun Liu, Maofa Jiang, Junjie Shi

**Affiliations:** ^1^Key Laboratory for Ecological Metallurgy of Multimetallic Mineral (Ministry of Education), Northeastern University, Shenyang, China; ^2^School of Metallurgy, Northeastern University, Shenyang, China; ^3^Hefei National Laboratory for Physical Sciences at the Microscale, CAS Key Laboratory of Materials for Energy Conversion, Division of Nanomaterials & Chemistry, Department of Materials Science and Engineering, University of Science and Technology of China, Hefei, China

**Keywords:** spent lithium-ion batteries, cathode and anode electrode, economic, cascade treatment, recovery and regeneration

## Abstract

It is of great economic, environmental and social benefit to discover harmless treatment and resource utilization options for spent lithium-ion batteries (LIBs), which contain a large proportion of valuable metal elements (e.g., Li, Ni, Co, Mn, Cu, and Al) and poisonous chemicals (e.g., lithium hexafluorophosphate and polyvinylidene fluoride). The present work summarized the leading technologies and hot issues in the disposal of spent LIBs from new energy vehicles. Moreover, development of the trend of innovative technologies for the recycling of spent LIBs is recommended.

## Introduction

**Figure 1:**
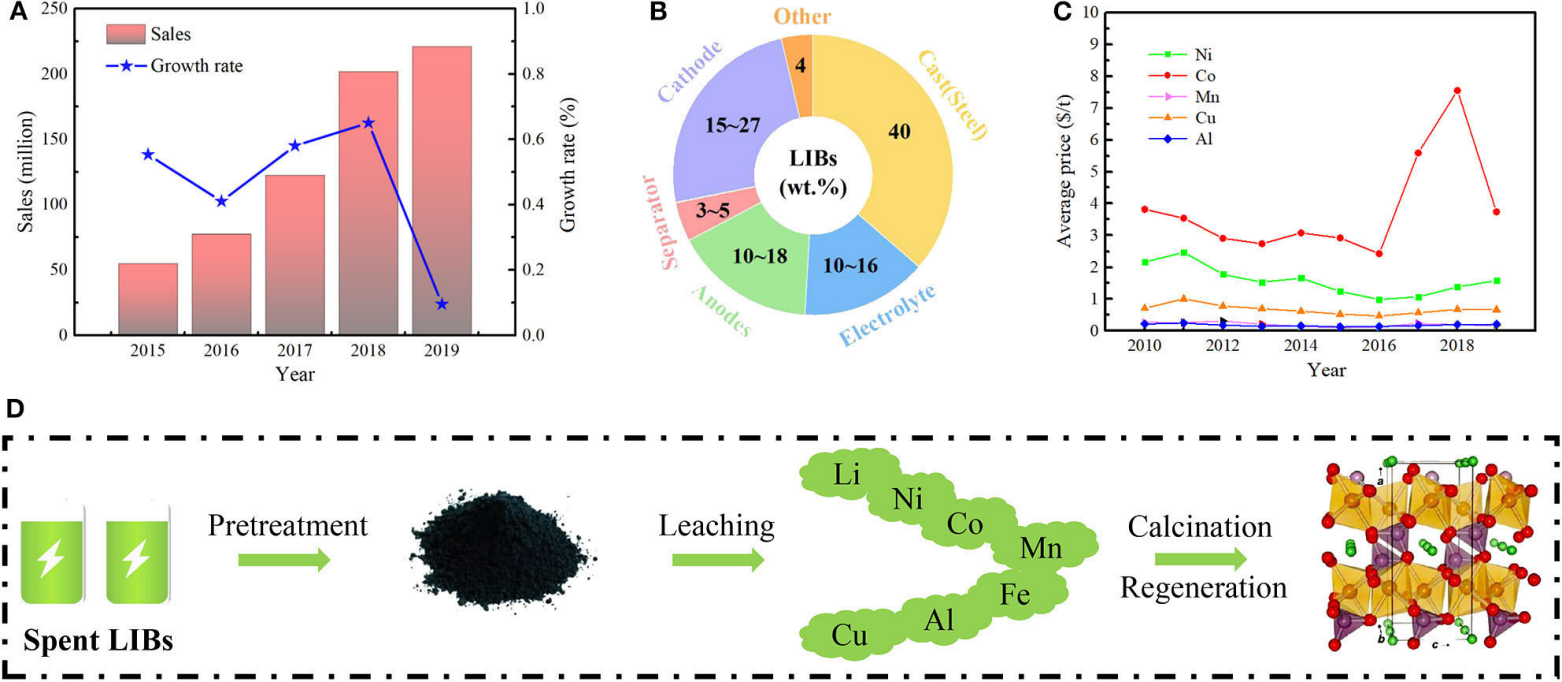
**(A)** Global new energy vehicle sales from 2015 to 2019. **(B)** Composition and proportion of each component of LIBs (Winter and Brodd, 2004). **(C)** Average prices of main metals in spent LIBs from 2010 to 2019. **(D)** Flowsheet for recycling of valuable metals from the spent LIBs. The data of **(A,C)** come from the public data collation.

Energy security, environmental pollution and climate deterioration have been regarded as the three major challenges restricting the world development since the industrial revolution. To alleviate environmental pollution and solve energy problems, the new energy vehicles have been vigorously promoted all around the world. The lithium-ion batteries (LIBs) have occupied the global battery market and have become the first choice of power battery due to the advantages of high power density, low self-discharge, high average output voltage, and long service life (Deng, 2015; Choi and Wang, 2018; Huang et al., 2018; Li et al., 2018) (Figure 1A). However, the internal structure of the LIBs could become irreversible after a hundred cycles of charging and discharging, which will block the diffusion channel of Li^+^, and ultimately leads to the inactivation and scrapping of LIBs. Therefore, the average life of LIBs is only 3 ~ 5 years (Palacin and Guibert, 2016). With the rapid increasing consuming of LIBs, the number of spent LIBs has also increased explosively worldwide. It is predicted that the number of spent LIBs in 2020 will surpass 25 billion units, i.e., 500 thousand tons (Zeng et al., 2014).

The spent LIBs are mainly composed of cathode and anode materials, electrolytes, diaphragms, binders, and shell (Winter and Brodd, 2004) (Figure 1B). If the spent LIBs are not handled properly, the electrolytes and diaphragms will cause fluorine and organic pollutions (Lv et al., 2017), and the cathode/anode materials could lead to a heavy metal pollution. From another point of view, the spent LIBs are also known as an “urban mine,” which contain carbonate organic solvents, lithium hexafluorophosphate and a large proportion of valuable metal elements (Li, Co, Ni, Cu, Fe, Al, etc.) (Meshram et al., 2014; Harper et al., 2019) (Figure 1C). Elements of Li, Co, and Ni in cathode materials account for 2 ~ 12%, 5 ~ 30%, and 0 ~ 10%, respectively. Cu and Al are mainly used for current collectors with contents of 7 ~ 17% and 3 ~ 10%, respectively, and the Fe in the outer shell is in the range of 0 ~ 25% (Lv et al., 2017; Choi and Wang, 2018; Huang et al., 2018). The contents of some valuable metals contained in spent LIBs are higher than their corresponding primary ores. In consequence, rational recycling, and regeneration of the spent LIBs is conducive to relieving the shortage of high-quality primary Li, Co, and Ni resources, as well as an important aspect of green and sustainable development of the new energy industry.

## Recycling and Regeneration Technologies

Recycling and regeneration technologies of spent LIBs can be divided into three steps (Joulié et al., 2014; Sa et al., 2015; Zhao et al., 2020): (1) Pretreatment, composed by two processes of primary and secondary processes (Yang et al., 2015). (2) Recycling of electrode materials, including hydrometallurgical, pyrometallurgical, and biological metallurgical methods, or their coupled methods (Nirmale et al., 2017; Winslow et al., 2018). (3) High-value regeneration of electrode materials, including the preparation of precursors from the separating and precipitating valuable metals by the methods of coprecipitation (Liu et al., 2018), sol-gel (Li et al., 2017), hydrothermal (Yang et al., 2015), and high temperature calcination (Figure 1D). Among the steps, the pretreatment acts as the foundation of the whole treatment process, and leaching of valuable metals is the premise to realize comprehensive recovery of metal components. The last step of regeneration is the core process, which could generate high value-added products from LIBs (Zheng et al., 2018).

### Recycling of Spent LIBs

The basic point of recycling and recovery of spent LIBs is to realize efficient extraction of the valuable metal elements. The hydrometallurgical process can recover and purify battery materials from the spent LIBs, and the reported leaching yields of Li, Ni, Co, and Mn were all more than 90% (Meshram et al., 2015; Liu et al., 2018). However, excessive consumption of acid and alkali will cause secondary pollution and easily corrode the equipment (Yao et al., 2018). The pyrometallurgical thermal reduction of spent LIBs can obtain a Ni-Co-Fe metal phase and a slag phase composed by the oxides of Li and Mn (Meshram et al., 2014), while the disadvantages of the high temperature (above 1,300°C), high energy consumption, high pollution and low extraction efficiency of lithium restrict its application. The biological metallurgy process has been regarded as the most environmentally favorable technique, which is achieved through the use of inorganic and organic acids produced by the metabolism of different microorganisms and strains to dissolve the spent LIBs materials. It was proposed that the metal recovery of 100% Cu, 100% Li, 77% Mn, 75% Al, 64% Co, and 54% Ni could be achieved via the biological method (Nazanin and Mousavi, 2017). However, this method is still in laboratory stage due to the problems of slow extracting speed, harsh requirements on the survival environment of strains, and long culture cycle of strains (Nazanin et al., 2018; Zhao et al., 2019).

### Regeneration of Spent LIBs

Throughout the current industrial recycling processes for spent LIBs, the selective separation of the associated components is still a bottleneck problem for cost saving and technology improvement, and the regeneration technologies of battery materials could solve this problem well. It utilizes the co-existence and collaborative extraction characteristics of valuable metal ions in the complex system to regenerate battery materials, which forms a closed-loop treatment that has great development potential.

To realize the high-value regeneration of valuable components recovered from spent LIBs, researchers have developed supporting technologies such as coprecipitation-calcination regeneration, sol-gel-calcination regeneration, hydrothermal-calcination regeneration, etc. Among which the coprecipitation approach is regarded as a promising method since valuable components could synergistically, uniformly, and compositely precipitate from the leachate of electrode materials. It was reported that battery materials prepared via coprecipitation method met the standards of commercial battery (The initial discharge specific capacity is 172.9 mAh·g^−1^) (Liu et al., 2018). However, a large number of alkali agents were inevitably consumed, and improper control of acidity of the solution would lead to the problems of precipitation, adsorption and agglomeration. The sol-gel method is to add an appropriate complexing agent to the leaching solution. After uniform mixing, hydrolysis, and polymerization, reactions occur forming a stable transparent sol system. Cathode material could be regenerated after dried and calcined treatments. The sol-gel method can avoid the use of a large amount of alkaline solution, and can also refine the material particles and improve the element uniformity. The electrochemical performances of the prepared battery material are equivalent to that of the commercial NCM cathodes (Li et al., 2017) (The initial discharge specific capacity is 149.8 mAh·g^−1^). However, large amounts of complexing agents and flocculants will be used to ensure the complete precipitation, which increases the cost of this process. The hydrothermal method is by the reaction of hydrolysis, polycondensation and dehydration under high temperature and high pressure conditions, which can effectively control the particle size of the product and improve the crystallinity of the product. The electrochemical performances of the battery material prepared by the hydrothermal method are equivalent to the raw materials synthesized under the same conditions (Yang et al., 2015) (The initial discharge specific capacity is 147.6 mAh·g^−1^). However, it has the disadvantages of a long reaction time, small sediment particles and difficult recovery. Moreover, it is difficult for the performance of the regenerated battery materials to meet the standards of commercial battery materials due to the uncontrollable composition and proportion of valuable components in the recovered product. All of these hinder the further development of recycling and regeneration of spent LIBs. How to improve the recycling efficiency and realize the high-value utilization of the recycled products is worthy of study by all spent LIBs recycling enterprises (Deng, 2015; Swain, 2017; Yang et al., 2018).

## Industrialization

Currently, there are a few large-scale enterprises that recycle LIBs in the world, e.g., Umicore, the subsidiary of Toshiba, TERUME, Sumitomo Metal Mining, INMETCO, Toxco, and AEA Technologies of the United Kingdom. However, the overall scale is small, and some key technologies need to be broken through. At present, some steps of regeneration technology of spent LIBs are mutually independent, integration designs for short processes that should be conducted by engineers. Furthermore, support equipment and recycling services need to innovate with the technology development. How to achieve the co-existence of spent LIBs and the environment, and how to dispose and recycle the spent LIBs effectively, have become an important issue facing the sustainable development of the battery industry (Swain, 2017; Zhang et al., 2018).

## Conclusions

The new energy vehicle industry is a strategic emerging industry in many countries, the recycling and regeneration of spent LIBs has become the bottleneck of its sustainable development. A general survey of the current recycling technology and industrial actuality shows that there are still many problems, e.g., the insufficient research on recycling mechanisms, immature directional conversion technology, incomplete utilization of integrated systems, backward technology and equipment level, low added value of recycled products, and serious secondary pollution. It is proposed that the key points and difficulties in the treatment of spent LIBs mainly exist in following four aspects: the cascade utilization of battery, the harmless disposal of electrolyte, the resource utilization of cathode and anode materials, and the recycling and regeneration of battery materials. The cascade utilization of battery is to apply the capacity attenuation to <80% to the national power grid, basic equipment and other fields that have relatively low battery requirements. When the capacity is <50%, follow-up recovery and regeneration processing is performed. Based on the comparative analysis of the research status of different treatment processes, it is clear that the key bottleneck restricting the industrial application and large-scale promotion of technologies lies in the following: (1) In the pre-treatment stage of spent LIBs, the low efficiency and mechanization automation degree of selective separation of electrode active substances hinder the further improvement of separation efficiency. (2) In the stage of recovering valuable components of battery materials, it is difficult to balance the leaching selectivity of electrode materials and the recovery efficiency. A large amount of acid and alkali is consumed in the process of treatment, the recovery process is complicated, and the integration level of each treatment is low. (3) In the stage of regenerated battery material, it is difficult to accurately control the valence state of the regenerated metals, and the performance of the regenerated material still needs to be further improved. Moreover, the overall production process is long and energy consumption is high.

In view of the above problems, researchers are suggested to carry out technological breakthroughs and theoretical innovations in the following aspects: (1) The new separation technology (such as intelligent vertical eddy current separation) can be adopted to realize the efficient and selective separation of each component of electrode material. (2) To solve the problem of high energy consumption in traditional pyrometallurgy process and large discharge of waste liquid from the hydrometallurgy process, low-temperature ammonium roasting technology can be served as a potential process to replace the existing high-temperature carbon thermal reduction and acid leaching. It can reduce the treatment temperature of spent LIBs, avoid the use of large amounts of strong acid-base and reducing agents, save energy, and reduce the generation of waste. (3) In the process of resource cascade recovery and harmless treatment of electrolytes, reasonable technology linking and process integration should be carried out. Develop the field auxiliary technology and strengthening agents to improve the recognition degree of target group reaction, to satisfy the requirements of short process integration and realize high-value utilization of resources. (4) For the battery material regeneration technology, new powder treatment technology (such as high-temperature ball milling) is considered to jointly realize the calcination, refinement, homogenization treatment, and precise control of valence of materials, so as to reduce the introduction of impurities.

## Author Contributions

QZ carried out the concepts and design of the article. WL provided literature rearch. LH carried out manuscript editing. CL, MJ, and JS performed manuscript review. All authors contributed to the article and approved the submitted version.

## Conflict of Interest

The authors declare that the research was conducted in the absence of any commercial or financial relationships that could be construed as a potential conflict of interest. The handling Editor declared a shared affiliation, though no other collaboration, with the authors QZ, LH, WL, CL, MJ, and JS.
